# Eight Patients With Pilonidal Carcinoma in One Decade—Is the Incidence Rising?

**DOI:** 10.7759/cureus.27054

**Published:** 2022-07-20

**Authors:** Mhd F Safadi, Khaldoun Ghareb, Ayham Daher, Marius Dettmer, Hadeel Shamma, Dietrich Doll

**Affiliations:** 1 Visceral, Thoracic and Vascular Surgery, University Hospital Carl Gustav Carus, Dresden, DEU; 2 Colorectal and Proctologic Surgery, Charme Day Surgery Center, Dubai, ARE; 3 General Surgery, Al-Assad University Hospital, Damascus, SYR; 4 Surgery, St. Marienhospital Vechta, Vechta, DEU; 5 Pathology, Klinikum Chemnitz, Chemnitz, DEU; 6 Surgery, University of the Witwatersrand, Johannesburg, ZAF

**Keywords:** social media communication, health care in crisis, squamous cell carcinoma of the skin, squamous cell carcinoma (scc), pilonidal cyst surgery, pilonidal malignancy, pilonidal carcinoma, pilonidal, chronic pilonidal sinus, pilonidal sinus surgery

## Abstract

Introduction: Carcinoma secondary to pilonidal disease is very rare with fewer than 130 reported cases so far. It is presumed that underreporting and underpublishing contribute to the low reported incidence.

Methods: A post was published on a closed Facebook group with about 30,000 Syrian doctors asking if anyone had ever seen a patient with pilonidal carcinoma before. The patients' data were collected retrospectively from the treating physicians.

Results: Between 2010 and 2019, we identified eight patients with pilonidal carcinoma. All patients were males with a mean age of 55.5 years. The mean interval between diagnosis of pilonidal disease and diagnosis of carcinoma was 6.9 years. A growing ulcer on the background of a pilonidal sinus disease was the presenting complaint in 50% of cases. Three patients were lost from follow-up after the diagnosis due to referral. All other five patients underwent surgical resection and three of them received postoperative chemoradiation. Four patients were followed for six months or longer: two died of metastases, one survived after recurrence and re-excision, and one survived with no recurrence.

Conclusion: This paper presents the largest cohort of pilonidal carcinoma so far and the first that describes the disease in the Syrian population. Due to underreporting, the real incidence of pilonidal carcinoma exceeds what is reported so far in the literature.

## Introduction

Carcinoma that emerges on a background of pilonidal disease is a very rare entity. Fewer than 130 cases were reported in the literature so far [1]. Although the calculated incidence varies between publications, it is estimated that malignant transformation occurs in about 0.1% of patients with the pilonidal disease [2,3].

The largest reported series about this disease was from Almeida-Gonçalves from Spain who treated seven patients with cryoablation and published his results in 2012 [4]. Another series from Macedonia reported five patients, four of whom developed metastases [5]. Most other reports described only one case per article.

In this paper, we report a cohort of eight Syrian patients with pilonidal sinus disease carcinoma (PSD-Ca), who were diagnosed and treated within one decade during the Syrian crisis. This is the largest cohort of this disease so far and the first one that describes pilonidal disease carcinoma in the Syrian population.

## Materials and methods

The cases were collected using communication with doctors on social media. We published a post on a closed Facebook group with about 30,000 Syrian doctors from all specialties including general practitioners, surgeons, pathologists, and oncologists. We asked if anyone had ever had a patient with carcinoma of the pilonidal sinus before. Within 48 hours, eight doctors commented that they had seen the disease before. In the comments, we asked them to supply detailed information through an online questionnaire.

To avoid misdiagnosing a primary squamous cell carcinoma of the gluteal cleft as a malignant transformation secondary to pilonidal sinus disease, all included patients were required to have previous symptoms that are typical for a pilonidal disease but are not seen with primary squamous cell carcinomas of the skin, such as a previous local abscess or the presence of draining sinuses. This was confirmed by the reporting doctors as most patients were previously known to them and some of them underwent previous operations.

The retrospectively collected data included the year of diagnosis, age, and gender of the patients, the interval between diagnosis of the pilonidal disease and carcinoma as well as any previous operations, presenting symptoms, preoperative investigations including biopsy and imaging, histological type, presence of regional or distant metastases, provided surgical therapy, administration of neoadjuvant or adjuvant therapy, follow-up period, and general course of the disease including recurrence.

Retrospective review of the patients’ files was not possible due to the interval since diagnosis as well as the internal/external migration of both doctors and patients because of the crisis, as presented in the discussion. We asked the participants to provide the data anonymously and to the best of their knowledge. To avoid double-reporting, we checked the details of each case such as the year of diagnosis and the patient’s characteristics. The data were tabularly organized and analyzed using Microsoft Excel (Microsoft Corp., Redmond, WA, USA).

## Results

**Figure 1:**
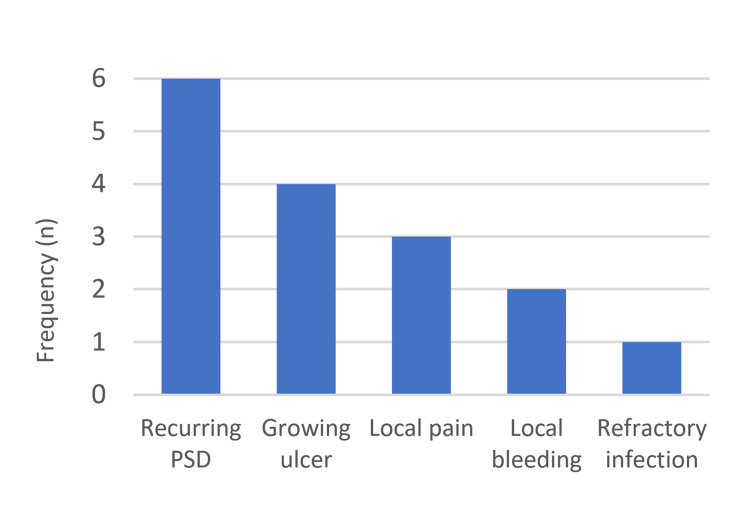
The presenting complaints The chart shows the main presenting complaints in the study group. Some patients had more than one complaint on presentation. PSD: Pilonidal sinus disease

Eight patients were diagnosed with PSD-Ca between 2010 and 2019 (Table 1). All patients were males with a median age of 57.5 years (mean±SD: 55.5±5.8 years, range: 45 to 60 years). The median interval between diagnosis of pilonidal disease and carcinoma was four years (mean±SD: 6.9±7.1 years, range: 1.5 to 20 years). Most of the patients were operated on once or twice before. In one patient, 13 operations were performed and varied from simple incision to resection with reconstruction. The most common complaint upon presentation was a growing ulcerated lesion on a background of chronic, non-healing pilonidal disease, which was reported in 50% of cases. The frequency of the other symptoms is illustrated in Figure 1.

**Table 1:** Overview of the patients in this study ID: Case identification number, PSD: Pilonidal sinus disease, SCC: Squamous cell carcinoma, LTFU: Lost to follow-up * These values may not be accurate and are estimated to the best knowledge of the reporting doctors.

ID	Year of diagnosis	Age at diagnosis (years)^*^	Interval from PSD to cancer (years)^*^	Histological type of the tumor	Follow-up period (months)^*^	Course of the disease and treatment
1	2010	50	5	SCC	24	Radical excision and flap reconstruction, no recurrence in the follow-up period
2	2012	55	2	SCC	LTFU	LTFU
3	2014	60	unknown	SCC	6	Radical excision and secondary healing, bilateral inguinal recurrence, death
4	2015	45	10	SCC	72	Local recurrence with multiple operations, no recurrence in the follow-up period
5	2016	60	unknown	SCC	LTFU	LTFU
6	2017	60	3	unknown	LTFU	LTFU
7	2017	60	20	SCC	1.5	LTFU
8	2019	52	1.5	SCC	12	Unilateral inguinal lymphadenopathy, iliac and paraaortic metastases, death

In the clinical examination, the maximal diameter of the lesions ranged from 2 cm to 10 cm. In one patient (case 3), the disease was so extended that it involved the buttock region bilaterally. A biopsy was performed preoperatively in four patients (50%). In two patients (25%), the resection was performed primarily without biopsy despite suspicion of malignancy, because the lesions were small and amenable to en-bloc resection. In two other patients (25%), the malignancy was an incidental finding in the histological study after resection. Squamous cell carcinoma (SCC) constituted the histological type in seven patients. In the eighth patient (case 6), the reporting doctor was not sure of the tumor type.

Due to the unavailability of medical services in the time of crisis, two of the eight patients were referred to larger hospitals in other cities for further investigations and treatment. Another patient preferred to travel abroad for treatment (case 2). These three patients were lost from follow-up early and after confirmation of the diagnosis. The other five patients could be followed by the reporting doceriod of 1.5 months and six years. Long-term survival was documented in only two patients. Of these two patients, the first (case 1) was treated with radical resection and flap reconstruction with no adjuvant therapy and was disease-free after two years. The other (case 4) underwent resection and coverage with a rotational flap. A later recurrence was treated with re-excision, and the patient was disease-free after six years.

Overview of the cases

Case 1

**Figure 2:**
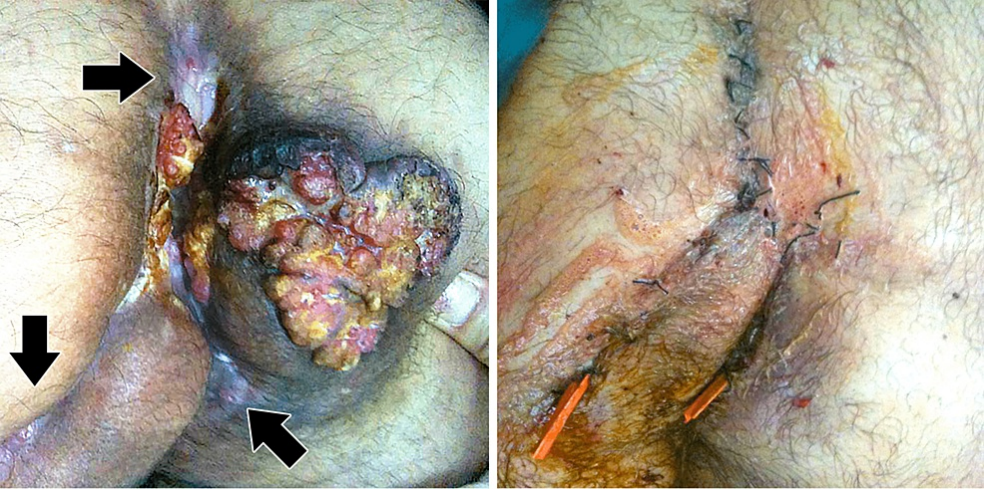
Images of patient 1 in the study Left: An ulcerated, exfoliated mass in the sacral region with dominant growth towards the right side. Diffuse scarring can be seen after two previous operations for pilonidal disease (arrows). Right: The same patient after tumor excision and reconstruction using rotational flaps.

A 50-year-old man presented with refractory pilonidal disease with occasional local bleeding and discomfort. His pilonidal sinus disease was known for about five years and was operated on twice. The examination showed a 10 cm ulcerated lesion (Figure 2, left). The patient underwent en-bloc resection of the tumor with the coccyx. The defect was reconstructed using rotational flaps (Figure 2, right). The histological examination showed a moderately differentiated squamous cell carcinoma (SCC). The patient was disease-free after two years.

Case 2

A 55-year-old man was operated on two years before presentation, where the lesion was resected but not sent for histological evaluation. The patient presented with a 5x3 cm recurrent lesion in the intergluteal cleft with an ulcerated growth. The biopsy confirmed the suspicion of SCC. However, the patient was not convinced arguing that “I have never heard that cancer can develop in a hair cyst!”. He chose to travel abroad to Iraq for further treatment and was lost from follow-up.

Case 3

A 60-year-old man presented with a large ulcerative lesion involving the whole buttock with bilateral inguinal lymphadenopathy. The biopsy confirmed SCC and the computed tomography showed paraaortic lymph node metastases. The extreme discomfort and local complaints remained despite radiotherapy, so a palliative resection with secondary wound care was offered. The patient died of metastases six months later.

*Case 4* 

A 45-year-old patient with chronic hepatitis C and a known pilonidal disease presented with a refractory lesion. The patient reported a noticeable history with 13 previous operations over 10 years, ranging from simple incision to resection with reconstruction. The current lesion was about 5x4 cm in maximal dimensions. The patient was treated with local excision and reconstruction using a rotational flap, and he refused the submission of the specimen for histological examination due to financial costs. A further operation was performed after six months and again after 12 months because of non-healing. Here, the histology revealed the presence of SCC. The patient received adjuvant chemoradiation. According to the treating doctor, the patient developed a local recurrence of the benign pilonidal disease, but without recurrence of malignancy after six years of follow-up.

*Case 5* 

A 60-year-old patient presented with a non-healing wound after operative treatment of a pilonidal cyst a few months before presentation. The examination showed an 8x5 cm lesion with ulceration, bleeding, and local extension to the anal region. The biopsy confirmed the suspicion of SCC. The patient was referred to another city for definite treatment, after which he was lost from follow-up.

Case 6

A 60-year-old patient presented with a recurrence of pilonidal disease after four operations over the last three years. The examination showed no ulceration but local scarring with multiple sinuses within an area of 10x7 cm. The lesion was found to be infiltrating the coccyx and was treated with resection and secondary wound healing. Histological examination showed malignancy and the patient was referred to another city for oncological counseling, after which he was lost from follow-up.

Case 7

A 60-year-old patient with a known PSD for about 20 years presented with a 6x4 cm growing ulcer in the sacral area. Initiated by high clinical suspicion, a tissue biopsy showed an SCC. Additional workup included coloscopy and computed tomography of the abdomen. The patient was treated with local resection and then referred to chemoradiation. The reporting doctor could not provide further follow-up results beyond six weeks after treatment.

Case 8

A 52-year-old patient presented with a 2x2 cm growing pilonidal lesion with local pain and recurrent infections. The lesion was resected and the defect was reconstructed using rotational flaps. The malignancy was an incidental finding on histological examination. A few months later, the patient returned with local recurrence as well as unilateral inguinal lymphadenopathy. The therapy included re-excision with chemoradiation. In the further follow-up, the computed tomography showed iliac and paraaortic lymph node metastases. The patient died of the metastatic disease about one year after diagnosis.

## Discussion

Although pilonidal disease carcinoma was reported in the literature since the beginning of the twentieth century, Weinstein et al. were the first authors who related it to the secondary squamous cell carcinoma described by Marjolin. They explicitly described its association with the chronic inflammatory process, stating that “chronic irritation and inflammation are favorable soils for the im­plantation of carcinoma cells” [6]. The time interval between the first manifestation of the pilonidal disease and carcinoma varies widely in the literature: from an initial presentation with malignancy to the development of cancer after 62 years of chronic disease [7]. On average, the latency period for the development of carcinoma equals about 25 years of untreated disease [8]. This coincides with the reported duration for the development of Marjolin’s ulcer which ranges from 11 to 75 years with an average of 30 years [9].

As seen in this study, SCC presents the most common histological type. The other reported types include verrucous carcinoma, basal cell carcinoma, mixed squamous and basal carcinoma, adenocarcinoma, and even rhabdomyosarcoma in one case [5,10]. The microscopic appearance of these tumors is characterized by malignant squamous epithelium with atypical cells that infiltrate the underlying layers [11]. Due to the associated chronic inflammation, it is typical to find areas of granulation, necrosis, and foreign body reactions with abundant inflammatory cells [12].

As the histological examination was not performed routinely in all patients after resection of an apparently naïve pilonidal lesion, failed postoperative healing with the development of chronic ulcer was the most prominent symptom. Other symptoms can include recurrent bleeding and growing mass [13]. Upon suspicion, biopsies should be obtained from the margin of the lesion [14]. The staging workup may include clinical examination (including the inguinal lymph nodes), rectosigmoidoscopy, and imaging studies such as an X-ray of the pelvic bones, pelvic magnetic resonance imaging, or computed tomography [13,15].

Surgical resection is the mainstay of treatment and includes radical resection of the tumor together with the involved structures. The resulting defect can be left for secondary healing or closed with flaps or skin grafts, either primarily or as a delayed procedure [16]. Abdominoperineal resection was performed for local invasion [17]. In locally advanced cases, neoadjuvant chemoradiation may be considered to downstage the tumor and decrease local recurrence rates [13]. Adjuvant chemotherapy is reserved for patients with regional or systematic metastases and its impact on the prognosis is not clear [3,18]. Local cryosurgery was also described by Almeida-Gonçalves in seven patients with excellent results but was not reproduced by other authors [4]. The prognosis of the disease is related to the stage at diagnosis, but the survival rate for five years is almost 55% to 61% [19].

In addition to the disease characteristics presented above, we would like to shed some light on other interesting points in this study. These include the potential increased incidence of PSD-Ca, the impact of the crisis on the health care service as well as scientific research, and the emerging role of social media in medical research.

Incidence of PSD-Ca

Since the reporting of the first case of PSD-Ca from Wolf in 1900 [20], about 130 cases were documented in the literature over the last 120 years. This equals roughly about one case per year worldwide, with more cases being published starting from the sixties of the twentieth century [1].

However, the detection of eight, previously unreported cases within one decade and from one country raises a lot of inquiries about the real incidence of this disease. These eight cases actually double the worldwide reported number of cases in the last decade. As early as 1963, Milch et al. suggested that the low incidence of this disease may be due to the lack of submission of tissue specimens for pathological examination or inadequate histological examination of the specimens [21]. In two of the eight patients, the malignancy was an incidental, unexpected finding. So, the routine submission of the resected specimen for pathology is highly recommended regardless of the age of the patient, as neither age nor disease duration is necessarily correlated with the malignant transformation [22].

Besides, we are faced with another factor that was highlighted by Dettmer et al. in 2021: it seems that PSD-Ca is extremely underreported and underpublished by the physicians [22], as these eight cases were not to be discovered and reported if the survey were not published on the Facebook group. Underreporting is not confined to surgeons, but also includes other specialists such as primary care physicians, oncologists, and pathologists.

Whether the incidence is rising or the previously unreported cases are simply being uncovered is a difficult issue to resolve. Although the pilonidal disease is very common in the surgical practice in Syria, there is no official data concerning its incidence among the population. So, it is not possible to estimate the incidence of malignant transformation due to the unavailability of data about the prevalence of the disease.

Impact of the crisis on the medical practice

As the respected reader may notice, the follow-up of the patients and the collection of data were inadequate, which presents the main limitation of this study. The Syrian crisis has impacted many social and financial aspects of life, including health care services. This impaired the access to medical care, data documentation, follow-up of patients, and ability to conduct meaningful clinical research.

On one hand, it is estimated that about half of healthcare facilities were plundered or destroyed due to combat during the crisis [23], which made the retrograde collection of patients’ data impossible. This also deprived thousands of people of access to health care and urged them to flee to other regions. Low follow-up rates due to rapid changes in demographics were already reported in other studies on Syrian patients during the crisis [24].

On the other hand, it is estimated that about 70% of healthcare providers migrated to other countries, which caused them to lose contact with their previous patients or workplaces [24]. Many of the physicians who reported the cases in this study no longer live in Syria. We believe that it is a great misfortune to spot such rare cases without being able to collect enough data or follow them up properly.

Role of social media in medical research

Although this topic falls outside the scope of the paper, we want to stress that communication with health care providers over social media enabled us to discover eight unreported cases of a very rare disease, which could have gone undetected. We believe that the role of social media in medical research should be better discussed, prompted, and regulated, as this medium may unveil hidden treasures in medical knowledge.

Social media has opened a new window for medical research and enabled investigators to reach out to an increasing range of data sources. There is no need to stress that social media communication has already found applications in multiple research domains such as crowdsourcing answers to clinical questions, recruitment of patients in clinical trials, and reaching social peer-support communities [25].

Advantages of seeking participating physicians using online channels may include outreaching a large number of physicians with little effort and enabling distant communication and interaction. However, the employment of social media in medical research is not without challenges. Among the most critical issues are ethical considerations such as written consent, patients’ privacy, and the accuracy of the provided information [25,26]. We have to acknowledge that the data compiled using this medium is far from complete or perfect. However, it was still valuable in expanding our view of pilonidal carcinoma.

## Conclusions

Carcinoma arising from longstanding pilonidal sinus disease may be far more common than the available literature suggests. This article presented a cohort of eight Syrian patients, which is the largest worldwide so far and the first one that documents the disease in the Syrian population. The Syrian crisis had a deep impact on the health care services, including early diagnosis and treatment as well as patients’ follow-up and data assembly for scientific purposes. We believe that the incidence of PSD-Ca should be investigated with more accuracy, especially in developed and underserved countries. Surgeons, pathologists, and oncologists are encouraged to report every case of the disease to identify its real incidence and improve therapy protocols.
